# Domain-level epitope mapping of polyclonal antibodies against HER-1 and HER-2 receptors using phage display technology

**DOI:** 10.1038/s41598-022-16411-z

**Published:** 2022-07-18

**Authors:** Dayana Pérez-Martínez, Yanelys Cabrera Infante, Belinda Sánchez Ramírez, Gertrudis Rojas

**Affiliations:** Center of Molecular Immunology, calle 216 esq 15, apartado 16040, Atabey, Playa, CP 11300 La Habana, Cuba

**Keywords:** Biochemistry, Biotechnology, Cancer, Immunology

## Abstract

HER-1 and HER-2 are tumor-associated antigens overexpressed in several epithelial tumors, and successfully targeted by therapeutic approaches against cancer. Vaccination with their recombinant extracellular domains has had encouraging results in the pre-clinical setting. As complex humoral responses targeting multiple epitopes within each antigen are the ultimate goal of such active immunotherapy strategies, molecular dissection of the mixture of antibody specificities is required. The current work exploits phage display of antigenic versions of HER-1 and HER-2 domains to accomplish domain-level epitope mapping. Recognition of domains I, III and IV of both antigens by antibodies of immunized mice was shown, indicating diverse responses covering a broad range of antigenic regions. The combination of phage display and site-directed mutagenesis allowed mutational screening of antigen surface, showing polyclonal antibodies’ recognition of mutated receptor escape variants known to arise in patients under the selective pressure of the anti-HER-1 antibody cetuximab. Phage-displayed HER domains have thus the potential to contribute to fine specificity characterization of humoral responses during future development of anti-cancer vaccines.

## Introduction

The family of Human Epidermal growth factor Receptors (HERs) includes four members: EGF-R/HER-1/ErbB1, HER-2/ErbB2, HER-3/ErbB3 and HER-4/ErbB4, which are transmembrane proteins involved in proliferation, survival and differentiation of epithelial cells^1^. These molecules have an extracellular domain (ECD) that interacts with ligands, a transmembrane domain, and a cytoplasmic domain involved in signal transduction^1^. The ECD is composed by four shorter domains with unique structural and functional features: domains I and III, with inherent ligand binding ability, domain II including a protruding arm for homo- or hetero-dimerization between HER molecules, and membrane-proximal domain IV^2^. Deregulation of the expression and function of HER family members is linked to cancer development, and these molecules are well established as tumor-associated clinically relevant targets^3^. HER-related malignancies comprise epithelial tumors of ovary, prostate, breast, head and neck, colon and lung^4–10^. Signaling cascades activated by HERs support proliferation, apoptosis evasion, angiogenesis, migration and metastasis of cancer cells^11^.

Monoclonal antibodies (mAbs) against HER family members such as cetuximab, nimotuzumab, panitumumab, trastuzumab and pertuzumab have achieved outstanding results in the clinical setting^12^. Powerful anti-tumor effects of these antibodies as ligand binding inhibitors and/or mediators of immune effector mechanisms have been shown. Experiments showing the advantages of combinations of anti-HER antibodies over the use of single antibodies indicated the importance of targeting more than one epitope^13–17^. Multi-specific polyclonal responses to vaccination of patients with whole HER ECDs would allow simultaneous targeting of a plethora of epitopes on the same antigen, thus producing natural antibody cocktails able to mediate different biological mechanisms, which should result in increased anti-tumor potency. HER-based vaccines are indeed being developed with promising results in animal models^18–20^. Optimized vaccination protocols could guarantee sustained levels of functional circulating antibodies, but could also be an attractive technical solution to circumvent treatment resistance, an already described limitation of mAb therapy. Tumors can evolve to the resistant state due to their intrinsic heterogeneity and the occurrence of in vivo selection of cells displaying loss-of-epitope mutated target variants that are no longer recognized by a given antibody^21–24^. The likelihood of tumor escape from diverse polyclonal responses should be diminished as compared to mono-specific mAb selective pressure.

Therefore, the evaluation of the outcomes of HER immunization procedures should not be limited to global antibody levels titration. Showing the diversity of polyclonal responses is mandatory, and the development of efficient methods to do that is a pre-requisite for development of such vaccines. Common epitope mapping techniques, based on short synthetic peptides reproducing the primary sequence of the antigen^25,26^ or libraries of phage-displayed random peptides^27^ that mimic antigen regions, have more chances of being useful to identify linear epitopes than conformational ones. Many relevant HER epitopes are conformation-sensitive and their formation is strongly dependent on the correct folding of large antigenic regions constrained by the presence of multiple disulfide bonds within each domain^28^. Recapitulation of isolated HER domains for epitope mapping has been achieved using yeast display, with the advantages associated to biosynthesis in an eukaryotic host^29^. Phage display, the oldest and most extended display platform^30^, has also been used for similar purposes. Phage display of large protein fragments, 100 amino acids (aa) long, has been proposed as an alternative to conventional peptide libraries in studies aimed at identifying conformational epitopes of HER-2^31^. Antigenic HER-1 domain III has been successfully displayed in our laboratory and used to map fine specificity of mAbs^28^.

The current work extended previous experiences to the display of antigenic versions of domains of both HER-1 and HER-2. These tools were useful to show the diversity of polyclonal antibody responses to HER-1 and HER-2 in a mice vaccination model. Domains I, III and IV of each antigen were recognized. The combination of phage display and site-directed mutagenesis allowed mutational screening of antigen surface, showing polyclonal antibodies’ recognition of mutated receptor escape variants known to arise in patients under the selective pressure of the anti-HER-1 antibody cetuximab. The phage display platform developed here for domain-level epitope mapping could aid to the characterization of humoral responses during future development of HER-based vaccines.

## Results

### HER-1 and HER-2 extracellular domains were individually displayed as PVIII fusion proteins on filamentous phage

**Figure 1:**
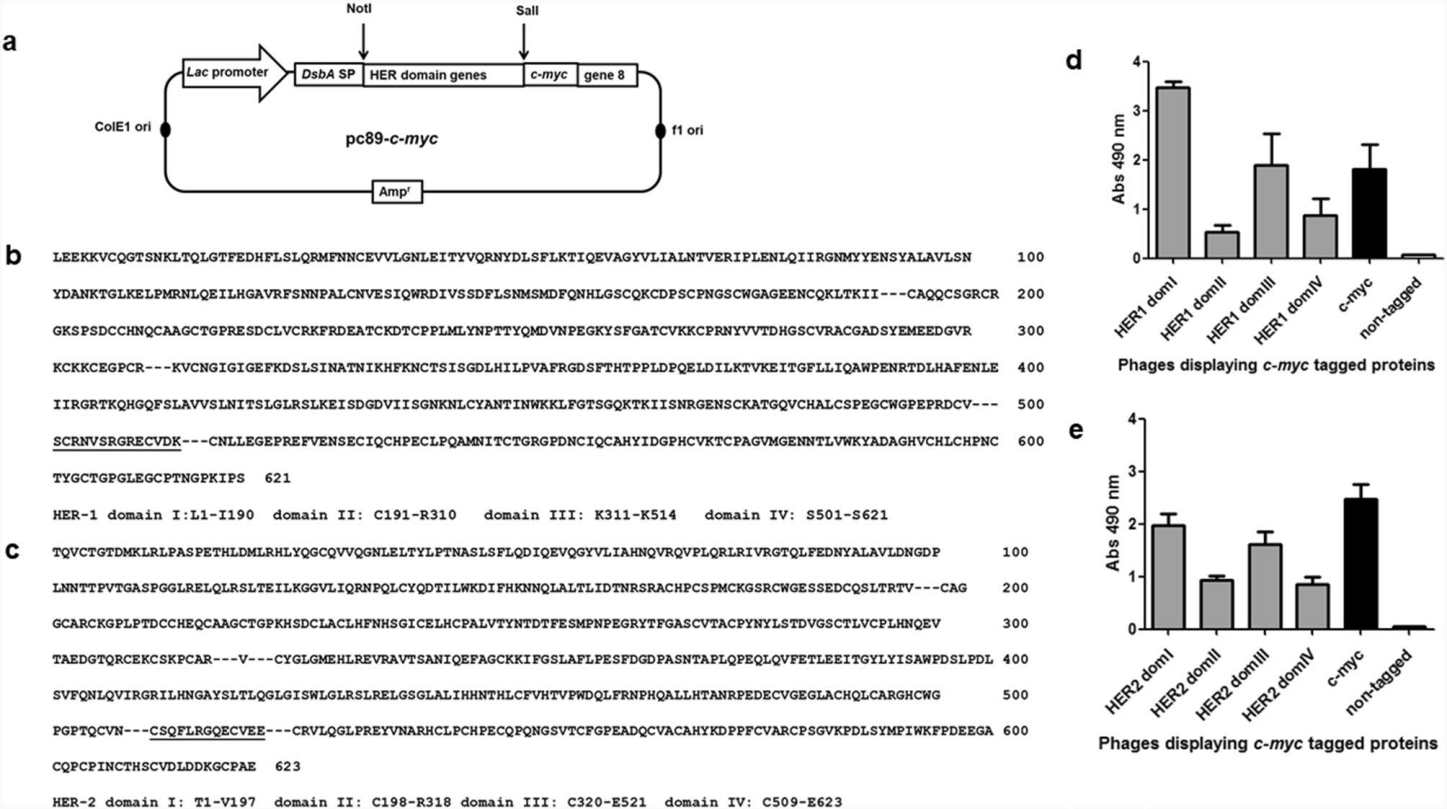
Display of HER-1/HER-2 domains on filamentous phages. Schematic representation of genetic constructs (**a**). Modified pC89-*c-myc* phagemid vector contains *Lac* promoter, phage and plasmid replication origins, an ampicillin resistance gene, and the genes coding for DsbA signal peptide, *c-myc* tag and filamentous phage PVIII. Genes coding for each HER domain were inserted between NotI and SalI restriction sites. HER-1 (**b**) and HER-2 (**c**) extracellular domain protein sequences and sequences of displayed domains are shown. Boundaries of the domains chosen for phage display are indicated with dotted lines. Two segments of 14 and 13 residues that are included by design in both the third and fourth domains of HER-1 and HER-2 respectively, appear underlined. ELISA recognition of HER-1 (**d**) and HER-2 (**e**) domains by the anti-*c-myc* tag antibody 9E10 is shown. Polyvinyl chloride microplates were coated with 9E10. Purified phages displaying domains of either HER-1 or HER-2 (5 × 10^10^ cfu/mL) were incubated on coated plates. Bound phages were detected with an anti-M13 antibody conjugated to horseradish peroxidase. Phages rescued from cells transformed with the empty pC89-*c-myc* and pC89 (untagged) vectors were used as positive and negative controls respectively.

Insertion of gene sequences coding for each of the four domains within the extracellular regions of either HER-1 or HER-2 near the 5' end of M13 gene 8 resulted in eight genetic constructs (Fig. 1a) suitable for multivalent display of the corresponding protein fragments (Fig. 1b,c). Recognition by 9E10 mAb, an antibody to the *c-myc* tag fused to displayed proteins in our system, showed the presence of every individual HER-1/HER-2 domain on phage surface after rescue with M13KO7 helper phage (Fig. 1d,e). As each construct codes for a single polypeptide comprising a signal peptide (removed by signal peptidase from the final protein), the foreign protein to be displayed, the c*-myc* tag and M13 PVIII, the presence of a reactive tag on phage surface implied that the whole in-frame polypeptide was expressed.

Quantitation of the display levels (a composite of the concentration of phage particles and the display levels of foreign protein per viral particle) on the basis of 9E10 binding using a standard curve of phage displaying *c-myc* tag alone, provided an estimation of the differences between phage preparations (Table 1). Domains II and IV were displayed at lower levels as compared to domains I and III. The difference was more remarkable in the case of HER-1.

**Table 1:** Display levels of the individual HER domains.

Antigen	Domain	Display levels (AU/mL)
HER-1	I	367.5 ± 4.8
	II	28.9 ± 3
	III	159.9 ± 12.8
	IV	49.8 ± 18.2
HER-2	I	273.9 ± 17.8
	II	105.8 ± 5.4
	III	245.2 ± 2.0
	IV	71.4 ± 14.3

Phages displaying every individual HER domain were rescued with M13KO7 helper phage and purified through polyethylene glycol precipitation. Each phage preparation was diluted and titrated on polyvinyl chloride microplates coated with the anti-*c-myc* tag antibody 9E10. Bound phages were detected with an anti-M13 antibody conjugated to horseradish peroxidase. The standard curve of phages displaying *c-myc* only with no fused protein was simultaneously evaluated and used to calculate the relative display levels for each preparation. A display level of 100 arbitrary units (AU)/mL was assumed for the undiluted standard. Numbers indicate the mean value ± standard deviation of three independent phage preparations displaying each domain.

**Figure 2:**
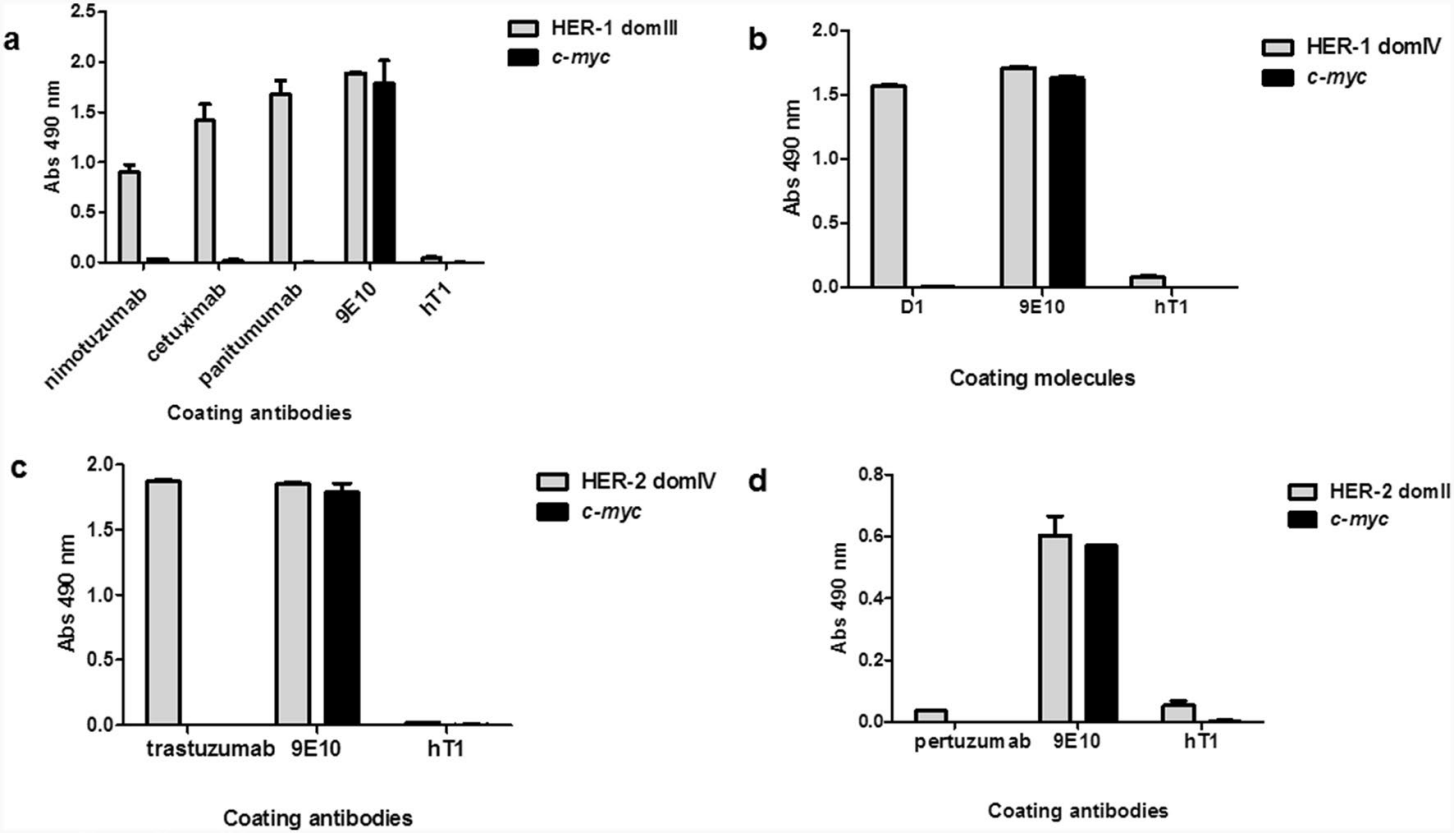
Recognition of phage-displayed domains of HER-1 and HER-2 by monoclonal antibodies in ELISA. Purified phages were incubated on polyvinyl chloride microtiter plates coated with monoclonal antibodies. Bound phages were detected with anti-M13 antibody conjugated to horseradish peroxidase. Humanized T1 antibody (hT1) was used as unrelated coating antibody to assess specificity. Coating 9E10 mAb recognizing all *c-myc* tagged proteins was used as positive control. Phages displaying *c-myc* only were also evaluated. Recognition of phage-displayed HER-1 domain III by nimotuzumab, cetuximab and panitumumab (**a**). Recognition of HER-1 domain IV by D1 (scFv)_2_-Fc molecule (**b**). Recognition of HER-2 domain IV by trastuzumab (**c**). Lack of recognition of HER-2 domain II by pertuzumab (**d**).

Monoclonal antibodies known to recognize specific domains of HER-1 and HER-2 were used as probes to confirm proper folding and antigenicity of the displayed proteins. Nimotuzumab, cetuximab and panitumumab were able to capture phage-displayed HER-1 domain III as expected (Fig. 2a). As all these antibodies target conformational epitopes, the above results point to a successful recapitulation of HER-1 domain III tri-dimensional structure on phage surface. Different degrees of reactivity correlate with relative affinities within this antibody panel. An additional anti-HER-1 molecule ((scFv)_2_-Fc format) named D1, previously constructed at our laboratory through single-chain Fv library screening against HER-1 whole ECD, showed specific recognition of domain IV (Fig. 2b), providing further support to the notion of epitope sharing between native HERs and their phage-displayed fragments.

In the case of HER-2, a first evidence of successful phage display came from recognition of domain IV by trastuzumab (Fig. 2c). In contrast, total failure of pertuzumab to recognize phage-displayed HER-2 domain II (Fig. 2d) showed that at least its target epitope was not properly displayed. Such a result highlighted the risk of incomplete recapitulation of the antigenic properties in some phage-displayed domains. Noteworthy, reactivity of all the commercial antibodies used as probes, including pertuzumab, was verified by ELISA on the corresponding soluble recombinant ECD in parallel with phage experiments (Supplemental Fig. S1).

**Figure 3:**
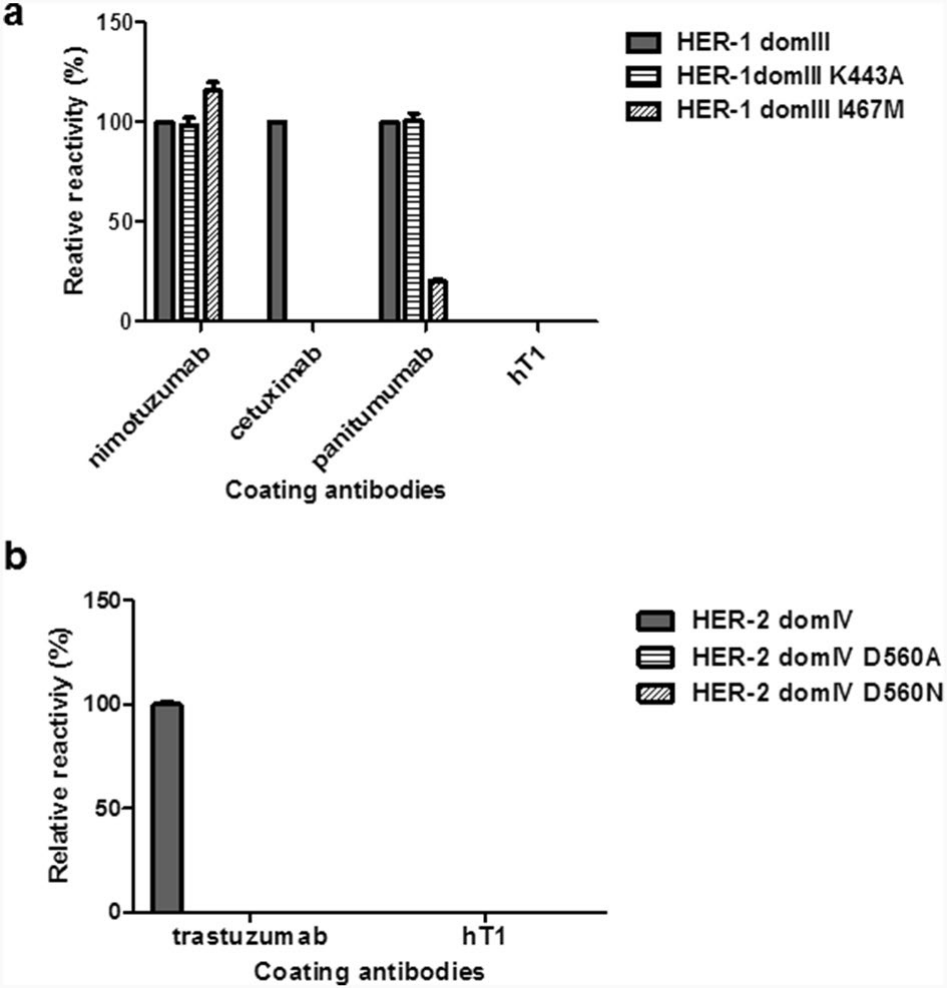
Recognition of mutated variants of phage-displayed domain III of HER-1 and domain IV of HER-2 by monoclonal antibodies. Purified phages displaying either wild-type (wt) or mutated HER domains were incubated on polyvinyl chloride microtiter plates coated with monoclonal antibodies. Bound phages were detected with an anti-M13 antibody conjugated to horseradish peroxidase. Relative reactivity (%) of each variant against a given antibody was calculated taking recognition of the wt non-mutated domain by the same antibody as the reference value (100%). Recognition of mutated variants of HER-1 domain III (**a**). Recognition of mutated variants of HER-2 domain IV (**b**).

Despite the possible loss of some epitopes, reliable reproduction of the original antigenic properties was further confirmed by the effects of selected mutations on binding. Specific loss of recognition upon the introduction of mutations affecting residues already known to be critical for cetuximab epitope formation^28^ (I467 and K443) was indeed observed for HER-1 domain III (Fig. 3a). The effect of K443A was restricted to cetuximab recognition, in agreement with the subtle differences between fine specificities of antibodies against HER-1 domain III already described^22,28^. On the other hand, the replacement I467M also caused an important reduction of recognition by panitumumab, which binds to a partially overlapping epitope as compared to cetuximab^22^ (Fig. 3a). Loss of recognition of HER-2 domain IV by trastuzumab upon the introduction of both a conservative (D560N) and a non-conservative (D560A) mutation at a position predicted to contribute to trastuzumab binding^32^ (Fig. 3b), provided further support to the idea of successful recapitulation of epitope fine details on phage particles.

### Polyclonal antibodies against HER-1 and HER-2 ECDs recognized phage displayed domains I, III and IV of both receptors

Mouse polyclonal antibodies against HER-1 and HER-2 ECDs were induced by immunization with each model vaccine antigen emulsified in Freund’s adjuvant. Antibody titers in the order of 10^–5^ were detected after the full immunization protocol. Specific recognition of the immunizing antigen by Protein A-purified antibodies obtained from ascitic fluid collected from hyper immune animals was confirmed before using them in phage ELISA.

**Figure 4:**
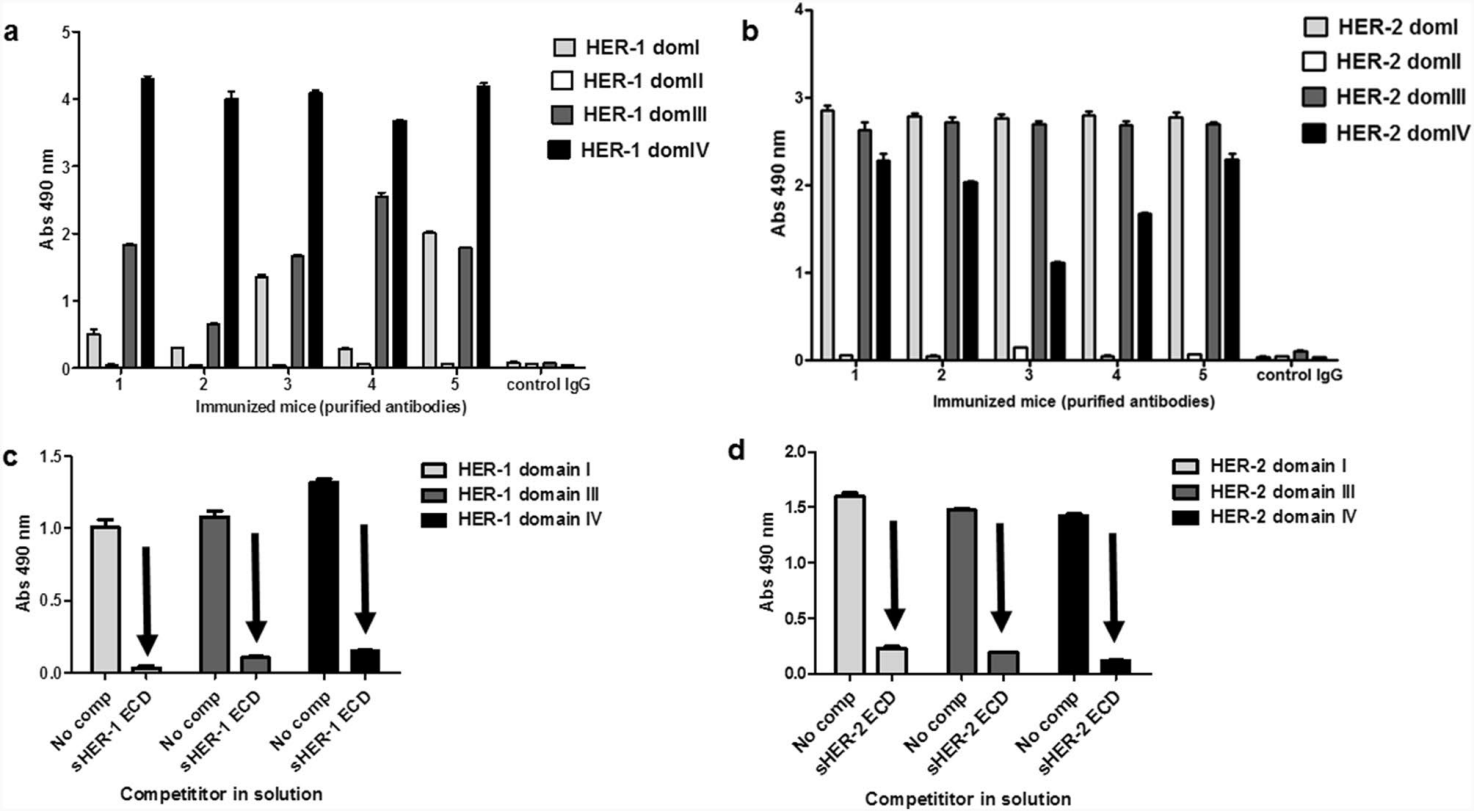
Domain recognition by antibodies elicited through immunization with HER-1/HER-2. Mice were immunized subcutaneously with a first dose of either HER-1 or HER-2 recombinant whole extracellular domain (ECD) emulsified in Complete Freund's Adjuvant, followed by three additional doses in Incomplete Freund's Adjuvant (IFA) every two weeks. Antibody-rich ascitic fluid was collected after intraperitoneal priming with IFA and myeloma inoculation, and IgG antibodies were purified by Protein A affinity chromatography. Reactivity against phage-displayed HER1 (**a**) and HER2 (**b**) domains was tested by ELISA on polyvinyl chloride microtiter plates coated with purified antibodies from each immunized mouse. Bound phage-displayed HER domains were detected with an anti-M13 mAb conjugated to horseradish peroxidase. Immunoglobulins from non-immunized mice were used as negative control. The specificity of recognition was assessed by competition ELISA. Phage-displayed individual domains of either HER-1 (**c**) or HER-2 (**d**) were either mixed with the whole recombinant ECD in solution (sECD) of the corresponding antigen or diluted without any competitor (No comp), and then added to microtiter plates coated with the pool of purified antibodies from immunized mice. Bound phage-displayed HER domains were detected with an anti-M13 mAb conjugated to horseradish peroxidase. Arrows indicate specific inhibition of recognition of phage-displayed HER domains by the competitor soluble ECD of each antigen.

Purified polyclonal antibodies from each individual mouse were shown to recognize specifically several phage-displayed domains of the corresponding immunizing antigen. Domains I, III and IV of HER-1 (Fig. 4a) and HER-2 (Fig. 4b) were recognized to different extents by antibodies from each immunized mouse. The fact that phage binding was completely abrogated by competition with soluble ECDs of both receptors (Fig. 4c,d) showed that it was not due neither to non-specific interactions mediated by large phage particles nor to specific recognition of phage components other than the displayed HER domains. The above results, taken as a whole, had dual implications. First, six of the eight phage-displayed individual domains were shown to recapitulate antigenic properties of the native receptors, now using polyclonal antibodies (potentially recognizing multiple epitopes) as probes. This result matches with data generated using the limited number of available monoclonal antibodies against three of the domains (see the previous section) and extends its scope. Second, antibody responses to both model antigens were shown to be diverse in terms of domain recognition, and phage ELISA was useful to segregate such a mixed reactivity.

Reasons for the lack of reactivity of domains II of both HER-1 and HER-2 could not be unequivocally determined. Previous failure of pertuzumab to recognize phage-displayed HER-2 domain II points to problems in its antigenicity, at least in the formation of some epitopes. Structural similarities between HER-1 and HER-2, and particularly between domains II of both antigens, could suggest similar defects in phage-displayed HER-1 domain II. Alternatively, lack of immunogenicity of domains II was not ruled out as a factor contributing to the observed results.

### Immunization-induced polyclonal antibodies react with clinically relevant mutated HER-1 variants

**Figure 5:**
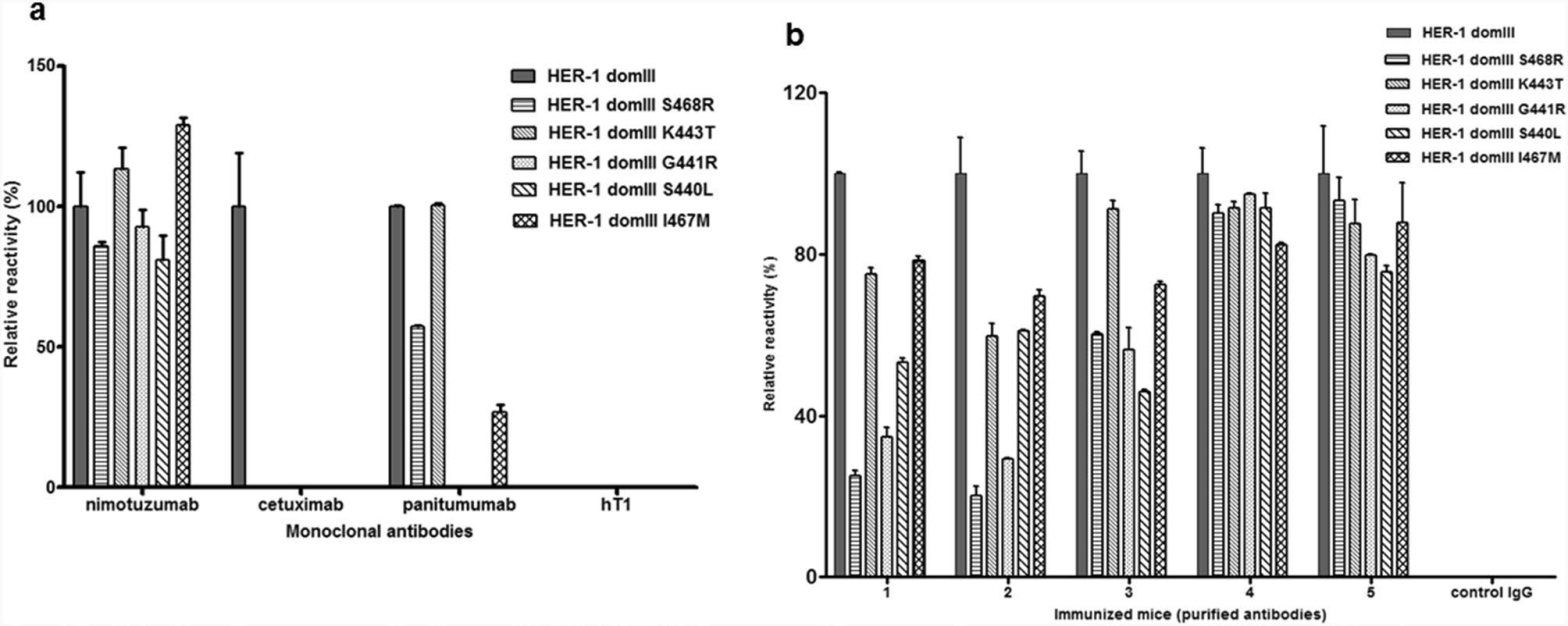
Reactivity of monoclonal and polyclonal antibodies against clinically relevant mutated variants of domain III of HER-1 conferring cetuximab treatment resistance. Recognition of phage-displayed domain III variants by monoclonal antibodies (**a**) and pooled polyclonal purified antibodies from HER-1 immunized mice (**b**) was evaluated through phage ELISA. The original phage-displayed HER-1 domain III and its mutated variants were incubated on microtiter plates coated with the different antibodies. Bound phages were detected with an anti-M13 mAb labeled with horseradish peroxidase. hT1 mAb and immunoglobulins from non-immunized mice (control IgG) were used as negative controls.

Antigen phage display, besides its usefulness to evaluate directly recognition of discrete antigenic regions, has a huge potential for high-throughput generation and screening of antigen variants. Mutations arising in HER-1 ECD domain III as a result of cetuximab therapy, already known to determine treatment resistance due to epitope loss despite antigen conservation in tumours, were thus explored through the combination of Kunkel mutagenesis^33^ and phage display. All the resulting mutated variants were not recognized at all by cetuximab, and some were also non-reactive with panitumumab, which recognizes a partially overlapping epitope^22^ (Fig. 5a). Highly conserved reactivity with nimotuzumab ruled out any gross defect in global folding and/or display of the mutated domains. Remarkably, higher recognition of these mutated HER-1 domain III versions by polyclonal antibodies (Fig. 5b) showed that multiepitopic recognition induced by immunization can compensate for specific epitope loss generated under antibody monotherapy selection pressure. In some cases, polyclonal responses seem to be completely non-sensitive to clinically relevant HER-1 mutations arising in patients. These findings highlight the usefulness of phage display of HER domains for mutational screening of the antigen surface and the advantages of polyclonal antibody responses over monoclonal antibodies to target antigens that are evolving in vivo.

## Discussion

Fine specificity characterization is key to understand the molecular bases of biological effects of antibodies. This task can be accomplished through a variety of well-established structural and functional methods^34,35^ in the case of monoclonal antibodies, for which a single binding interface is the subject of study. On the other hand, mapping humoral responses to vaccination is more challenging because of the involvement of a complex mixture of multiple antibody specificities, which should be simultaneously detected by suitable epitope mapping methods. Such techniques are usually based on dissection of global responses into individual reactivities against discrete parts of the target antigen. The most common approaches to do that are based on either short synthetic peptides recapitulating the primary sequence^36^ of a protein antigen or phage-displayed peptides known as mimotopes resembling antigen portions^37^. These techniques usually lead to the discovery of linear epitopes formed by residues that are contiguous or relatively close in the primary sequence and rather independent on global antigen folding.

HER family antigens have complex tertiary structures stabilized by multiple disulfide bonds^2^, and antibodies against them often target non-linear epitopes composed of amino acids located in distant regions of the sequence that form surface clusters in the tri-dimensional structure upon folding^28,38^. Mapping approaches based on large antigen fragments from them are thus likely to be best suited. Successful phage display of complete HER-1 domain III retaining its antigenicity was previously achieved in our laboratory, and used to delineate very detailed functional maps of the epitopes of anti-HER-1 monoclonal antibodies^28^. Results described here extend the scope to this strategy to other domains of both HER-1 and HER-2 and to a different field of application: mapping of polyclonal responses to vaccination with the whole ECDs. The idea behind the current work is related, although not identical, to phage display of large protein fragments already applied to HER-2^31^. Manipulating whole domains, naturally evolved as structural and functional independent protein building blocks, should contribute to recapitulation of the native antigenicity in such fragments. The precise boundaries between domains can be controversial and the design was fine-tuned following visual inspection of the 3D structure of HER-1 and HER-2 (PDB codes 1YY9 and 1N8Z). The N-terminal regions of Cys-rich domains (II and IV) were appended to domains I and III respectively in our phage-displayed versions, due to their tight packaging with such domains in both native structures. The addition of the N-terminal region of HER-1 domain IV had been previously shown to improve domain III expression and folding^39^. There was minimal overlap between domains (only 14/13 aa were shared between phage-displayed domains III and IV of HER-1/HER-2) to allow segregating the humoral responses against each of them.The choice of a pVIII-based multivalent display system^27^ contrasts with our previous experience (monovalent display on pIII) and its rationale was aimed at facilitating detection of subdominant antibody specificities under-represented within the mixture through avidity effects.

Detection of reactivity against domains I, III and IV of every immunizing antigen has implications to define the landscape of potential biological effects of vaccination. HER-1 domain III is indeed the target of very effective therapeutic mAbs that work by blocking receptor-ligand interactions (cetuximab, nimotuzumab and panitumumab) and precluding the receptor to adopt the active dimerization-ready conformation^28,38^. As ligand binding site involves both domain I and III^40^, antibodies against domain I can have similar functions. No ligands have been described yet for HER-2, which is in the extended conformation without the need for ligand binding^2^. The effects of antibodies to HER-2 domains I and III are thus more difficult to envision, although by virtue of their binding specificity they can theoretically affect this natural dimerization-prone conformation. HER recognition by antibodies, besides direct effects on ligand binding, can recruit immune effectors to attack tumor cells directly. Antibodies against the membrane-proximal domain IV of both antigens (highly prevalent in every immunized animal) can be particularly important for that effect, as it has been reported that membrane proximity of the target epitope can have a positive influence on antibody-dependent cell cytotoxicity^41^.

Failure of polyclonal antibodies to recognize domains II of any of the antigens can result from an aberrant protein folding of the displayed proteins. The designed domains II of HER-1 and HER-2 are rich in cysteines and require the formation of nine disulfide bridges each to stabilize the structure formed by loops and random coils. This can complicate their expression as recombinant proteins. Recombinant HER-2 domain II have had severe antigenicity problems even when produced in mammalian cell-based systems^42^ which are much closer to a natural biosynthesis context of human antigens than phage production. The presence of disulfide bonds, although can clearly contribute to display difficulties as it has been reported for other proteins^43^, does not rule out every possibility of successful display. Domains IV, very similar to domains II in structure and cysteine-rich composition (eight disulfide bonds each), were displayed in antigenic form, as shown through strong recognition by both monoclonal and polyclonal antibodies. The chosen display system was designed to achieve a quick co-translational secretion (through the use of a DsbA signal peptide) to the periplasmic compartment where disulfide bridge formation can readily occur^44^. It is possible that, besides proper disulfide bonds’ formation, structural stabilization by flanking domains is required for correct folding of domains II. In fact, fusion to domains I or III has been used in previous studies to achieve domain II expression^42,45^. Refining domain II display by testing alternative fusion protein designs, other expression conditions and the use of chaperones^46,47^ remains as a relevant goal, as dimerization arm (a critical moiety for receptor activation), is located in this region. Recognition of HER-2 domain II is indeed the basis of therapeutic effect of pertuzumab. Detecting antibodies against domain II, if they exist, is thus necessary to complete the functional landscape of possible biological effects of vaccination. Additional experiments are thus required to distinguish between a technical display failure and the inability to elicit antibodies against domain II with the experimental immunization protocols we used, which is also a valid possibility.

Even though the use of phage-displayed domains was aimed at dissecting antibody specificity at domain-level against whole domains rather than revealing fine details of individual epitopes, mutational screening of the displayed domains opens the possibility to answer relevant questions regarding mutated receptor variants. Growing body of evidence supports the notion of tumors evolving in vivo under the selective pressure of therapeutic antibodies targeting a given epitope. This kind of selection ends with treatment resistant tumors that sometimes lose expression of the target antigen, but can also display mutated antigen variants able to mediate the original biological functions without keeping the specific target epitope. This phenomenon has been well documented during the course of HER-1-directed therapies^21–24^. One of the potential advantages of vaccination responses targeting multiple epitopes is the ability to circumvent resistance against a single mono-specific agent. Recognition of a panel of phage-displayed HER-1 domain III variants having mutations in a cluster of residues located in the cetuximab binding interface^22^ (already known to confer resistance to cetuximab treatment) provided experimental support to this idea.

The usefulness of phage-displayed HER domains developed here was assessed with available mAbs and with polyclonal antibodies derived from a simple experimental immunization protocol with model antigens in Freund’s adjuvant. Once the antigenicity of several domains was shown, they are available as tools for the molecular dissection of humoral responses with optimized vaccination strategies, in both pre-clinical and clinical scenario.

## Methods

### Antigens

Soluble versions of recombinant HER-1 (aa 1–621) and HER-2 (aa 1–623) ECDs were provided by the group of Therapeutic Vaccines from the Center of Molecular Immunology (CIM), Cuba.

### Monoclonal antibodies

Nimotuzumab (TheraCIM, hR3) and itolizumab (non-related anti-CD6 antibody, also known as hT1) were obtained from CIMAB S.A., Cuba.

Trastuzumab (Herceptin), pertuzumab (Perjeta), cetuximab (Erbitux) and panitumumab (Vectibix) were also obtained from commercial sources (Roche, Genentech, Merck KGaA and AMGen respectively).

9E10 anti-c-myc tag monoclonal antibody was purchased from CIGB (Sancti Spiritus, Cuba).

D1 fusion protein comprising an anti-HER-1 domain IV scFv fused to a human Fc domain was produced at the Protein Engineering laboratory of the Center of Molecular Immunology, Cuba.

Reactivity of monoclonal antibodies against HER-1/HER-2 ECDs was verified by indirect ELISA on antigen-coated microtitration plates.

### Generation and purification of polyclonal antibodies against HER-1 and HER-2

Two goups of five female SPF BALB/c Ann healthy mice each (12–18 weeks of age, 18-20 g), provided by the National Center for Laboratory Animals Production (CENPALAB, Cuba), were immunized four times every two weeks with HER-1 ECD (200 µg/animal) or of HER-2 (100 µg/animal). Antigens were emulsified in Complete Freund’s adjuvant for the first dose and Incomplete Freund’s adjuvant (IFA) for the subsequent immunizations. Induction of specific antibodies was shown by ELISA on microtitration plates coated with the immunizing antigen. Mice were intraperitoneally primed with IFA two weeks after the last immunization, and mouse P3X63-Ag8.653 (American Type Culture Collection, CRL-8375) myeloma cells (10^6^ cells/animal) were inoculated in the peritoneal cavity five days later. After ten days, ascitic fluid was collected. Ascites was also obtained from five non-immunized animals with the same characteristics (control group). IgG antibodies from either immunized or non-immunized animals ascitic fluid were purified by Protein-A affinity chromatography.

For the experiments described above, fifteen mice were used in total (five animals/group). This was considered the minimal suitable number of animals to characterize the antibody responses against the immunizing antigens, given the individual variability of immune responses. Mice were allocated to each group by simple randomization. There were five mice/cage. Before the experiments, mice were kept for seven days at CIM animal care unit (acclimatization period). The animals were maintained all the time at CIM animal care unit at 20–25 °C, 60 ± 5% relative humidity, with light/darkness cycles of 12 h. Food and water were administered ad libitum. At the end of the experiments, the animals were sacrificed.

### Phage display and site-directed mutagenesis of HER-1 and HER-2 domains

The genes coding for the four domains of HER-1 and HER-2 ECDs (flanked by NotI and SalI restriction sites) were amplified by PCR and cloned into pc89-*c-myc* phagemid vector (Fig. 1), inserted near to the 5’ end of the M13 PVIII-coding gene. The sequences of every insert were confirmed by Macrogen, Korea. The list of full DNA and protein sequences corresponding to each genetic construct (from Dsba signal peptide to the end of M13 PVIII) is shown in supplemental information. Phage particles displaying the domains were rescued with M13KO7 helper phage and purified by PEG-NaCl precipitation following established procedures^48^.

Mutated variants of genes coding for HER domains were generated by Kunkel mutagenesis using the above described genetic constructs as templates, and antisense mutagenic oligonucleotides (CIGB, Cuba)^48^. Sequence correctness was confirmed by DNA sequencing (Microsynth, Germany). Full DNA and protein sequences corresponding to each mutated genetic construct (from Dsba signal peptide to the end of M13 PVIII) are shown in supplemental information. Phage particles displaying mutated variants of the domains were rescued as described above.

### Phage ELISA to determine relative display levels

The amounts of phage-displayed domains were determined by ELISA, measuring recognition of *c-myc* tag (fused to all displayed proteins) on 9E10-coated microtiter plates, using established procedures^48^. A standard curve of phages displaying *c-myc* alone was taken as reference, assuming the presence of 100 arbitrary display units/mL in the undiluted preparation. The relative display levels of HER domains in each preparation were calculated by interpolation from the standard curve.

### Phage ELISA to assess recognition of phage-displayed HER domains

Polyvinyl chloride microtiter plates were coated overnight at 4 °C with 10 μg/mL of monoclonal or polyclonal antibodies diluted in phosphate buffered saline (PBS). Plates were blocked 1 h at room temperature (RT) with 4% (w:v) skim milk in PBS (M-PBS). Purified phages displaying HER-1/HER-2 domains or its mutated variants (diluted in M-PBS) were added to the plates and incubated during 1 h at RT. Plates were washed with 0.1% Tween 20 (v:v) in PBS (PBS-T). An anti-M13 mAb conjugated to horseradish peroxidase (HRP, GE Healthcare, USA), appropriately diluted in M-PBS, was added. Plates were incubated 1 h at RT and washed as described with PBS-T. Substrate solution (500 μg/ml ortho-phenylenediamine and 0.015% hydrogen peroxide in 0.1 mol/L citrate–phosphate buffer, pH 5.0) was added. The reaction was stopped 15 min later with sulfuric acid (2.5 mol/L). The absorbance at 490 mm was determined with a microplate reader. In order to normalize the amounts of different phage-displayed domains within each experiment, equivalent concentrations (in display units/mL, calculated as described in the previous section) were used. For some experiments, samples were normalized on the bases of the total amount of phage particles (measured as colony forming units, cfu).

To perform phage competition ELISA, 20 µg/mL of HER-1/HER-2 soluble ECD were mixed with the diluted phage samples before incubation on coated/blocked plates.

#### Animal experiments’ statement

Protocols involving living animals were approved by the Institutional Committee of Laboratory Animal Care and Use (CICUAL) at the Center of Molecular Immunology. Animal experiments were performed in accordance with the relevant national and international guidelines and regulations. These experiments are reported here according to ARRIVE guidelines.

## Supplementary Information

Supplementary Information.

## Data Availability

All data generated and analysed during the current study are included in the published article and its supplementary information file. Any additional information is available from the corresponding author upon request.
