# Supplementary material for: Domain-level epitope mapping of polyclonal antibodies against HER-1 and HER-2 receptors using phage display technology
Source: Sci Rep. 2022 Jul 18;12:12268. doi: 10.1038/s41598-022-16411-z (PMC9293994; doi:10.1038/s41598-022-16411-z)
Supplement: Supplementary file 1 — Supplementary Information. [file 41598_2022_16411_MOESM1_ESM.pdf]

Supplemental information

Title:

Domain-level epitope mapping of polyclonal antibodies against HER-1 and HER-2 receptors using phage display technology

Authors:

Dayana Pérez-Martínez, Yanelys Cabrera Infante, Belinda Sánchez Ramírez, Gertrudis Rojas*

Author affiliation:

Center of Molecular Immunology; calle 216 esq 15, apartado 16040, Atabey, Playa, CP 11300, La Habana, Cuba

*Correspondence should be addressed to G.R.: grojas@cim.sld.cu

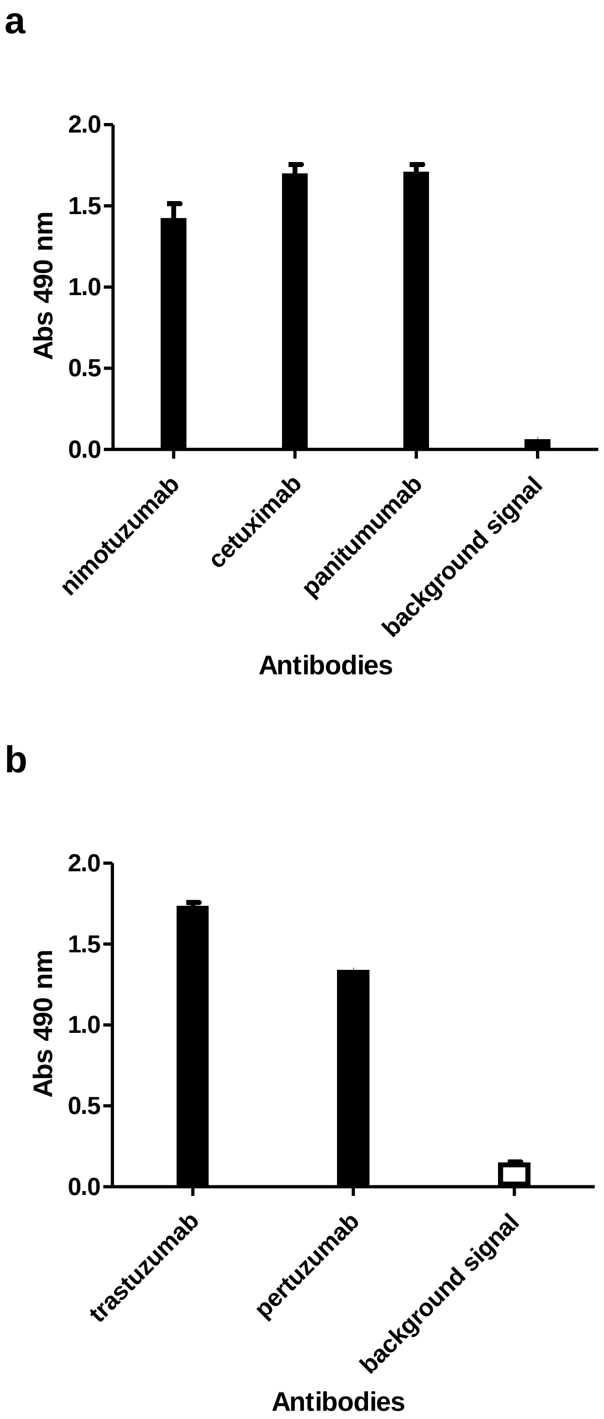

Supplememental Figure 1. Reactivity of commercial antibodies against HER-1 and HER-2. Polyvinyl chloride microtitration plates were coated with either HER-1 (a) or HER-2 (b) recombinant extracellular domains. Monoclonal antibodies (1 µg/mL) were incubated on coated plates and detected with an anti-human IgG antibody conjugated to horseradish peroxidase.

List of full DNA and deduced protein sequences of genetic constructs for phage display (from DsbA signal peptide to the end of M13 PVIII)

Color code:

DsbA signal peptide sequence

M13 PVIII sequence

Sequences of HER-1/HER-2 domains

***c-myc* tag sequence**

NotI (GCGGCCGC)and SalI (GTCGAC) restriction sites

Changes introduced by site-directed mutagenesis on HER domains are underlined.

- Sequence 1: HER-1domain I

M K K I W L A L A G L V L A F S A S A A E

ATGAAAAAGATTTGGCTGGCGCTGGCTGGTTTAGTTTTAGCGTTTAGCGCAAGTGCAGCTGAG

G A A A L E E K K V C Q G T S N K L T Q L

GGTGCGGCCGCCCTGGAGGAAAAGAAAGTTTGCCAAGGCACGAGTAACAAGCTCACGCAGTTG

G T F E D H F L S L Q R M F N N C E V V L

GGCACTTTTGAAGATCATTTTCTCAGCCTCCAGAGGATGTTCAATAACTGTGAGGTGGTCCTT

G N L E I T Y V Q R N Y D L S F L K T I Q

GGGAATTTGGAAATTACCTATGTGCAGAGGAATTATGATCTTTCCTTCTTAAAGACCATCCAG

E V A G Y V L I A L N T V E R I P L E N L

GAGGTGGCTGGTTATGTCCTCATTGCCCTCAACACAGTGGAGCGAATTCCTTTGGAAAACCTG

Q I I R G N M Y Y E N S Y A L A V L S N Y

CAGATCATCAGAGGAAATATGTACTACGAAAATTCCTATGCCTTAGCAGTCTTATCTAACTAT

D A N K T G L K E L P M R N L Q E I L H G

GATGCAAATAAAACCGGACTGAAGGAGCTGCCCATGAGAAATTTACAGGAAATCCTGCATGGC

A V R F S N N P A L C N V E S I Q W R D I

GCCGTGCGGTTCAGCAACAACCCTGCCCTGTGCAACGTGGAGAGCATCCAGTGGCGGGACATA

V S S D F L S N M S M D F Q N H L G S C Q

GTCAGCAGTGACTTTCTCAGCAACATGTCGATGGACTTCCAGAACCACCTGGGCAGCTGCCAA

K C D P S C P N G S C W G A G E E N C Q K

AAGTGTGATCCAAGCTGTCCCAATGGGAGCTGCTGGGGTGCAGGAGAGGAGAACTGCCAGAAA

L T K I I V D D P G E Q K L I S E E D L N

CTGACCAAAATCATCGTCGACGATCCGGGGGAACAAAAACTCATCTCAGAAGAGGATCTGAAT

G D P A K A A F D S L Q A S A T E Y I G Y

GGAGATCCCGCAAAAGCGGCCTTTGACTCCCTGCAAGCCTCAGCGACCGAATATATCGGTTAT

A W A M V V V I V G A T I G I K L F K K F

GCGTGGGCGATGGTTGTTGTCATTGTCGGCGCAACTATCGGTATCAAGCTGTTTAAGAAATTC

T S K A S

ACCTCGAAAGCAAGC

- Sequence 2: HER-1 domain II

M K K I W L A L A G L V L A F S A S A A E

ATGAAAAAGATTTGGCTGGCGCTGGCTGGTTTAGTTTTAGCGTTTAGCGCAAGTGCAGCTGAG

G A A A C A Q Q C S G R C R G K S P S D C

GGTGCGGCCGCCTGTGCCCAGCAGTGCTCCGGGCGCTGCCGTGGCAAGTCCCCCAGTGACTGC

C H N Q C A A G C T G P R E S D C L V C R

TGCCACAACCAGTGTGCTGCAGGCTGCACAGGCCCCCGGGAGAGCGACTGCCTGGTCTGCCGC

K F R D E A T C K D T C P P L M L Y N P T

AAATTCCGAGACGAAGCCACGTGCAAGGACACCTGCCCCCCACTCATGCTCTACAACCCCACC

T Y Q M D V N P E G K Y S F G A T C V K K

ACGTACCAGATGGATGTGAACCCCGAGGGCAAATACAGCTTTGGTGCCACCTGCGTGAAGAAG

C P R N Y V V T D H G S C V R A C G A D S

TGTCCCCGTAATTATGTGGTGACAGATCACGGCTCGTGCGTCCGAGCCTGTGGGGCCGACAGC

Y E M E E D G V R K C K K C E G P C R V D

TATGAGATGGAGGAAGACGGCGTCCGCAAGTGTAAGAAGTGCGAAGGGCCTTGCCGCGTCGAC

D P G E Q K L I S E E D L N G D P A K A A

GATCCGGGGGAACAAAAACTCATCTCAGAAGAGGATCTGAATGGAGATCCCGCAAAAGCGGCC

F D S L Q A S A T E Y I G Y A W A M V V V

TTTGACTCCCTGCAAGCCTCAGCGACCGAATATATCGGTTATGCGTGGGCGATGGTTGTTGTC

I V G A T I G I K L F K K F T S K A S

ATTGTCGGCGCAACTATCGGTATCAAGCTGTTTAAGAAATTCACCTCGAAAGCAAGC

- Sequence 3: HER-1domain III

M K K I W L A L A G L V L A F S A S A A E

ATGAAAAAGATTTGGCTGGCGCTGGCTGGTTTAGTTTTAGCGTTTAGCGCAAGTGCAGCTGAG

G A A A K V C N G I G I G E F K D S L S I

GGTGCGGCCGCCAAAGTGTGTAACGGAATAGGTATTGGTGAATTTAAAGACTCACTCTCCATA

N A T N I K H F K N C T S I S G D L H I L

AATGCTACGAATATTAAACACTTCAAAAACTGCACCTCCATCAGTGGCGATCTCCACATCCTG

P V A F R G D S F T H T P P L D P Q E L D

CCGGTGGCATTTAGGGGTGACTCCTTCACACATACTCCTCCTCTGGATCCACAGGAACTGGAT

I L K T V K E I T G F L L I Q A W P E N R

ATTCTGAAAACCGTAAAGGAAATCACAGGGTTTTTGCTGATTCAGGCTTGGCCTGAAAACAGG

T D L H A F E N L E I I R G R T K Q H G Q

ACGGACCTCCATGCCTTTGAGAACCTAGAAATCATACGCGGCAGGACCAAGCAACATGGTCAG

F S L A V V S L N I T S L G L R S L K E I

TTTTCTCTTGCAGTCGTCAGCCTGAACATAACATCCTTGGGATTACGCTCCCTCAAGGAGATA

S D G D V I I S G N K N L C Y A N T I N W

AGTGATGGAGATGTGATAATTTCAGGAAACAAAAATTTGTGCTATGCAAATACAATAAACTGG

K K L F G T S G Q K T K I I S N R G E N S

AAAAAACTGTTTGGGACCTCCGGTCAGAAAACCAAAATTATAAGCAACAGAGGTGAAAACAGC

C K A T G Q V C H A L C S P E G C W G P E

TGCAAGGCCACAGGCCAGGTCTGCCATGCCTTGTGCTCCCCCGAGGGCTGCTGGGGCCCGGAG

P R D C V S C R N V S R G R E C V D K V D

CCCAGGGACTGCGTCTCTTGCCGGAATGTCAGCCGAGGCAGGGAATGCGTGGACAAGGTCGAC

D P G E Q K L I S E E D L N G D P A K A A

GATCCGGGGGAACAAAAACTCATCTCAGAAGAGGATCTGAATGGAGATCCCGCAAAAGCGGCC

F D S L Q A S A T E Y I G Y A W A M V V V

TTTGACTCCCTGCAAGCCTCAGCGACCGAATATATCGGTTATGCGTGGGCGATGGTTGTTGTC

I V G A T I G I K L F K K F T S K A S

ATTGTCGGCGCAACTATCGGTATCAAGCTGTTTAAGAAATTCACCTCGAAAGCAAGC

- Sequence 4: HER-1 domain IV

M K K I W L A L A G L V L A F S A S A A E

ATGAAAAAGATTTGGCTGGCGCTGGCTGGTTTAGTTTTAGCGTTTAGCGCAAGTGCAGCTGAG

G A A A S C R N V S R G R E C V D K C N L

GGTGCGGCCGCCTCTTGCCGGAATGTCAGCCGAGGCAGGGAATGCGTGGACAAGTGCAACCTT

L E G E P R E F V E N S E C I Q C H P E C

CTGGAGGGTGAGCCAAGGGAGTTTGTGGAGAACTCTGAGTGCATACAGTGCCACCCAGAGTGC

L P Q A M N I T C T G R G P D N C I Q C A

CTGCCTCAGGCCATGAACATCACCTGCACAGGACGGGGACCAGACAACTGTATCCAGTGTGCC

H Y I D G P H C V K T C P A G V M G E N N

CACTACATTGACGGCCCCCACTGCGTCAAGACCTGCCCGGCAGGAGTCATGGGAGAAAACAAC

T L V W K Y A D A G H V C H L C H P N C T

ACCCTGGTCTGGAAGTACGCAGACGCCGGCCATGTGTGCCACCTGTGCCATCCAAACTGCACC

Y G C T G P G L E G C P T N G P K I P S V

TACGGATGCACTGGGCCAGGTCTTGAAGGCTGTCCAACGAATGGGCCTAAGATCCCGTCCGTC

D D P G E Q K L I S E E D L N G D P A K A

GACGATCCGGGGGAACAAAAACTCATCTCAGAAGAGGATCTGAATGGAGATCCCGCAAAAGCG

A F D S L Q A S A T E Y I G Y A W A M V V

GCCTTTGACTCCCTGCAAGCCTCAGCGACCGAATATATCGGTTATGCGTGGGCGATGGTTGTT

V I V G A T I G I K L F K K F T S K A S

GTCATTGTCGGCGCAACTATCGGTATCAAGCTGTTTAAGAAATTCACCTCGAAAGCAAGC

- Sequence 5: HER-2 domain I

M K K I W L A L A G L V L A F S A S A A E

ATGAAAAAGATTTGGCTGGCGCTGGCTGGTTTAGTTTTAGCGTTTAGCGCAAGTGCAGCTGAG

G A A A T Q V C T G T D M K L R L P A S P

GGTGCGGCCGCCACCCAAGTGTGCACCGGCACAGACATGAAGCTGCGGCTCCCTGCCAGTCCC

E T H L D M L R H L Y Q G C Q V V Q G N L

GAGACCCACCTGGACATGCTCCGCCACCTCTACCAGGGCTGCCAGGTGGTGCAGGGAAACCTG

E L T Y L P T N A S L S F L Q D I Q E V Q

GAACTCACCTACCTGCCCACCAATGCCAGCCTGTCCTTCCTGCAGGATATCCAGGAGGTGCAG

G Y V L I A H N Q V R Q V P L Q R L R I V

GGCTACGTGCTCATCGCTCACAACCAAGTGAGGCAGGTCCCACTGCAGAGGCTGCGGATTGTG

R G T Q L F E D N Y A L A V L D N G D P L

CGAGGCACCCAGCTCTTTGAGGACAACTATGCCCTGGCCGTGCTAGACAATGGAGACCCGCTG

N N T T P V T G A S P G G L R E L Q L R S

AACAATACCACCCCTGTCACAGGGGCCTCCCCAGGAGGCCTGCGGGAGCTGCAGCTTCGAAGC

L T E I L K G G V L I Q R N P Q L C Y Q D

CTCACAGAGATCTTGAAAGGAGGGGTCTTGATCCAGCGGAACCCCCAGCTCTGCTACCAGGAC

T I L W K D I F H K N N Q L A L T L I D T

ACGATTTTGTGGAAGGACATCTTCCACAAGAACAACCAGCTGGCTCTCACACTGATAGACACC

N R S R A C H P C S P M C K G S R C W G E

AACCGCTCTCGGGCCTGCCACCCCTGTTCTCCGATGTGTAAGGGCTCCCGCTGCTGGGGAGAG

S S E D C Q S L T R T V V D D P G E Q K L

AGTTCTGAGGATTGTCAGAGCCTGACGCGCACTGTCGTCGACGATCCGGGGGAACAAAAACTC

I S E E D L N G D P A K A A F D S L Q A S

ATCTCAGAAGAGGATCTGAATGGAGATCCCGCAAAAGCGGCCTTTGACTCCCTGCAAGCCTCA

A T E Y I G Y A W A M V V V I V G A T I G

GCGACCGAATATATCGGTTATGCGTGGGCGATGGTTGTTGTCATTGTCGGCGCAACTATCGGT

I K L F K K F T S K A S

ATCAAGCTGTTTAAGAAATTCACCTCGAAAGCAAGC

- Sequence 6: HER-2 domain II

M K K I W L A L A G L V L A F S A S A A E

ATGAAAAAGATTTGGCTGGCGCTGGCTGGTTTAGTTTTAGCGTTTAGCGCAAGTGCAGCTGAG

G A A A C A G G C A R C K G P L P T D C C

GGTGCGGCCGCCTGTGCCGGTGGCTGTGCCCGCTGCAAGGGGCCACTGCCCACTGACTGCTGC

H E Q C A A G C T G P K H S D C L A C L H

CATGAGCAGTGTGCTGCCGGCTGCACGGGCCCCAAGCACTCTGACTGCCTGGCCTGCCTCCAC

F N H S G I C E L H C P A L V T Y N T D T

TTCAACCACAGTGGCATCTGTGAGCTGCACTGCCCAGCCCTGGTCACCTACAACACAGACACG

F E S M P N P E G R Y T F G A S C V T A C

TTTGAGTCCATGCCCAATCCCGAGGGCCGGTATACATTCGGCGCCAGCTGTGTGACTGCCTGT

P Y N Y L S T D V G S C T L V C P L H N Q

CCCTACAACTACCTTTCTACGGACGTGGGATCCTGCACCCTCGTCTGCCCCCTGCACAACCAA

E V T A E D G T Q R C E K C S K P C A R V

GAGGTGACAGCAGAGGATGGAACACAGCGGTGTGAGAAGTGCAGCAAGCCCTGTGCCCGAGTC

D D P G E Q K L I S E E D L N G D P A K A

GACGATCCGGGGGAACAAAAACTCATCTCAGAAGAGGATCTGAATGGAGATCCCGCAAAAGCG

A F D S L Q A S A T E Y I G Y A W A M V V

GCCTTTGACTCCCTGCAAGCCTCAGCGACCGAATATATCGGTTATGCGTGGGCGATGGTTGTT

V I V G A T I G I K L F K K F T S K A S

GTCATTGTCGGCGCAACTATCGGTATCAAGCTGTTTAAGAAATTCACCTCGAAAGCAAGC

- Sequence 7: HER-2 domain III

M K K I W L A L A G L V L A F S A S A A E

ATGAAAAAGATTTGGCTGGCGCTGGCTGGTTTAGTTTTAGCGTTTAGCGCAAGTGCAGCTGAG

G A A A C Y G L G M E H L R E V R A V T S

GGTGCGGCCGCCTGCTATGGTCTGGGCATGGAGCACTTGCGAGAGGTGAGGGCAGTTACCAGT

A N I Q E F A G C K K I F G S L A F L P E

GCCAATATCCAGGAGTTTGCTGGCTGCAAGAAGATCTTTGGGAGCCTGGCATTTCTGCCGGAG

S F D G D P A S N T A P L Q P E Q L Q V F

AGCTTTGATGGGGACCCAGCCTCCAACACTGCCCCGCTCCAGCCAGAGCAGCTCCAAGTGTTT

E T L E E I T G Y L Y I S A W P D S L P D

GAGACTCTGGAAGAGATCACAGGTTACCTATACATCTCAGCATGGCCGGACAGCCTGCCTGAC

L S V F Q N L Q V I R G R I L H N G A Y S

CTCAGCGTCTTCCAGAACCTGCAAGTAATCCGGGGACGAATTCTGCACAATGGCGCCTACTCG

L T L Q G L G I S W L G L R S L R E L G S

CTGACCCTGCAAGGGCTGGGCATCAGCTGGCTGGGGCTGCGCTCACTGAGGGAACTGGGCAGT

G L A L I H H N T H L C F V H T V P W D Q

GGACTGGCCCTCATCCACCATAACACCCACCTCTGCTTCGTGCACACGGTGCCCTGGGACCAG

L F R N P H Q A L L H T A N R P E D E C V

CTCTTTCGGAACCCGCACCAAGCTCTGCTCCACACTGCCAACCGGCCAGAGGACGAGTGTGTG

G E G L A C H Q L C A R G H C W G P G P T

GGCGAGGGCCTGGCCTGCCACCAGCTGTGCGCCCGAGGGCACTGCTGGGGTCCAGGGCCCACC

Q C V N C S Q F L R G Q E C V E E V D D P

CAGTGTGTCAACTGCAGCCAGTTCCTTCGGGGCCAGGAGTGCGTGGAGGAAGTCGACGATCCG

G E Q K L I S E E D L N G D P A K A A F D

GGGGAACAAAAACTCATCTCAGAAGAGGATCTGAATGGAGATCCCGCAAAAGCGGCCTTTGAC

S L Q A S A T E Y I G Y A W A M V V V I V

TCCCTGCAAGCCTCAGCGACCGAATATATCGGTTATGCGTGGGCGATGGTTGTTGTCATTGTC

G A T I G I K L F K K F T S K A S

GGCGCAACTATCGGTATCAAGCTGTTTAAGAAATTCACCTCGAAAGCAAGC

- Sequence 8: HER-2 domain IV

M K K I W L A L A G L V L A F S A S A A E

ATGAAAAAGATTTGGCTGGCGCTGGCTGGTTTAGTTTTAGCGTTTAGCGCAAGTGCAGCTGAG

G A A A C S Q F L R G Q E C V E E C R V L

GGTGCGGCCGCCTGCAGCCAGTTCCTTCGGGGCCAGGAGTGCGTGGAGGAATGCCGAGTACTG

Q G L P R E Y V N A R H C L P C H P E C Q

CAGGGGCTCCCCAGGGAGTATGTGAATGCCAGGCACTGTTTGCCGTGCCACCCTGAGTGTCAG

P Q N G S V T C F G P E A D Q C V A C A H

CCCCAGAATGGCTCAGTGACCTGTTTTGGACCGGAGGCTGACCAGTGTGTGGCCTGTGCCCAC

Y K D P P F C V A R C P S G V K P D L S Y

TATAAGGACCCTCCCTTCTGCGTGGCCCGCTGCCCCAGCGGTGTGAAACCTGACCTCTCCTAC

M P I W K F P D E E G A C Q P C P I N C T

ATGCCCATCTGGAAGTTTCCAGATGAGGAGGGCGCATGCCAGCCTTGCCCCATCAACTGCACC

H S C V D L D D K G C P A E V D D P G E Q

CACTCCTGTGTGGACCTGGATGACAAGGGCTGCCCCGCCGAGGTCGACGATCCGGGGGAACAA

K L I S E E D L N G D P A K A A F D S L Q

AAACTCATCTCAGAAGAGGATCTGAATGGAGATCCCGCAAAAGCGGCCTTTGACTCCCTGCAA

A S A T E Y I G Y A W A M V V V I V G A T

GCCTCAGCGACCGAATATATCGGTTATGCGTGGGCGATGGTTGTTGTCATTGTCGGCGCAACT

I G I K L F K K F T S K A S

ATCGGTATCAAGCTGTTTAAGAAATTCACCTCGAAAGCAAGC

- Sequence 9: HER-1 domain III **S440L**

M K K I W L A L A G L V L A F S A S A A E

ATGAAAAAGATTTGGCTGGCGCTGGCTGGTTTAGTTTTAGCGTTTAGCGCAAGTGCAGCTGAG

G A A A K V C N G I G I G E F K D S L S I

GGTGCGGCCGCCAAAGTGTGTAACGGAATAGGTATTGGTGAATTTAAAGACTCACTCTCCATA

N A T N I K H F K N C T S I S G D L H I L

AATGCTACGAATATTAAACACTTCAAAAACTGCACCTCCATCAGTGGCGATCTCCACATCCTG

P V A F R G D S F T H T P P L D P Q E L D

CCGGTGGCATTTAGGGGTGACTCCTTCACACATACTCCTCCTCTGGATCCACAGGAACTGGAT

I L K T V K E I T G F L L I Q A W P E N R

ATTCTGAAAACCGTAAAGGAAATCACAGGGTTTTTGCTGATTCAGGCTTGGCCTGAAAACAGG

T D L H A F E N L E I I R G R T K Q H G Q

ACGGACCTCCATGCCTTTGAGAACCTAGAAATCATACGCGGCAGGACCAAGCAACATGGTCAG

F S L A V V S L N I T S L G L R S L K E I

TTTTCTCTTGCAGTCGTCAGCCTGAACATAACATCCTTGGGATTACGCTCCCTCAAGGAGATA

S D G D V I I L G N K N L C Y A N T I N W

AGTGATGGAGATGTGATAATTCTGGGAAACAAAAATTTGTGCTATGCAAATACAATAAACTGG

K K L F G T S G Q K T K I I S N R G E N S

AAAAAACTGTTTGGGACCTCCGGTCAGAAAACCAAAATTATAAGCAACAGAGGTGAAAACAGC

C K A T G Q V C H A L C S P E G C W G P E

TGCAAGGCCACAGGCCAGGTCTGCCATGCCTTGTGCTCCCCCGAGGGCTGCTGGGGCCCGGAG

P R D C V S C R N V S R G R E C V D K V D

CCCAGGGACTGCGTCTCTTGCCGGAATGTCAGCCGAGGCAGGGAATGCGTGGACAAGGTCGAC

D P G E Q K L I S E E D L N G D P A K A A

GATCCGGGGGAACAAAAACTCATCTCAGAAGAGGATCTGAATGGAGATCCCGCAAAAGCGGCC

F D S L Q A S A T E Y I G Y A W A M V V V

TTTGACTCCCTGCAAGCCTCAGCGACCGAATATATCGGTTATGCGTGGGCGATGGTTGTTGTC

I V G A T I G I K L F K K F T S K A S

ATTGTCGGCGCAACTATCGGTATCAAGCTGTTTAAGAAATTCACCTCGAAAGCAAGC

- Sequence 10: HER-1 domain III **G441R**

M K K I W L A L A G L V L A F S A S A A E

ATGAAAAAGATTTGGCTGGCGCTGGCTGGTTTAGTTTTAGCGTTTAGCGCAAGTGCAGCTGAG

G A A A K V C N G I G I G E F K D S L S I

GGTGCGGCCGCCAAAGTGTGTAACGGAATAGGTATTGGTGAATTTAAAGACTCACTCTCCATA

N A T N I K H F K N C T S I S G D L H I L

AATGCTACGAATATTAAACACTTCAAAAACTGCACCTCCATCAGTGGCGATCTCCACATCCTG

P V A F R G D S F T H T P P L D P Q E L D

CCGGTGGCATTTAGGGGTGACTCCTTCACACATACTCCTCCTCTGGATCCACAGGAACTGGAT

I L K T V K E I T G F L L I Q A W P E N R

ATTCTGAAAACCGTAAAGGAAATCACAGGGTTTTTGCTGATTCAGGCTTGGCCTGAAAACAGG

T D L H A F E N L E I I R G R T K Q H G Q

ACGGACCTCCATGCCTTTGAGAACCTAGAAATCATACGCGGCAGGACCAAGCAACATGGTCAG

F S L A V V S L N I T S L G L R S L K E I

TTTTCTCTTGCAGTCGTCAGCCTGAACATAACATCCTTGGGATTACGCTCCCTCAAGGAGATA

S D G D V I I S R N K N L C Y A N T I N W

AGTGATGGAGATGTGATAATTTCACGTAACAAAAATTTGTGCTATGCAAATACAATAAACTGG

K K L F G T S G Q K T K I I S N R G E N S

AAAAAACTGTTTGGGACCTCCGGTCAGAAAACCAAAATTATAAGCAACAGAGGTGAAAACAGC

C K A T G Q V C H A L C S P E G C W G P E

TGCAAGGCCACAGGCCAGGTCTGCCATGCCTTGTGCTCCCCCGAGGGCTGCTGGGGCCCGGAG

P R D C V S C R N V S R G R E C V D K V D

CCCAGGGACTGCGTCTCTTGCCGGAATGTCAGCCGAGGCAGGGAATGCGTGGACAAGGTCGAC

D P G E Q K L I S E E D L N G D P A K A A

GATCCGGGGGAACAAAAACTCATCTCAGAAGAGGATCTGAATGGAGATCCCGCAAAAGCGGCC

F D S L Q A S A T E Y I G Y A W A M V V V

TTTGACTCCCTGCAAGCCTCAGCGACCGAATATATCGGTTATGCGTGGGCGATGGTTGTTGTC

I V G A T I G I K L F K K F T S K A S

ATTGTCGGCGCAACTATCGGTATCAAGCTGTTTAAGAAATTCACCTCGAAAGCAAGC

- Sequence 11: HER-1 domain III **K443A**

M K K I W L A L A G L V L A F S A S A A E

ATGAAAAAGATTTGGCTGGCGCTGGCTGGTTTAGTTTTAGCGTTTAGCGCAAGTGCAGCTGAG

G A A A K V C N G I G I G E F K D S L S I

GGTGCGGCCGCCAAAGTGTGTAACGGAATAGGTATTGGTGAATTTAAAGACTCACTCTCCATA

N A T N I K H F K N C T S I S G D L H I L

AATGCTACGAATATTAAACACTTCAAAAACTGCACCTCCATCAGTGGCGATCTCCACATCCTG

P V A F R G D S F T H T P P L D P Q E L D

CCGGTGGCATTTAGGGGTGACTCCTTCACACATACTCCTCCTCTGGATCCACAGGAACTGGAT

I L K T V K E I T G F L L I Q A W P E N R

ATTCTGAAAACCGTAAAGGAAATCACAGGGTTTTTGCTGATTCAGGCTTGGCCTGAAAACAGG

T D L H A F E N L E I I R G R T K Q H G Q

ACGGACCTCCATGCCTTTGAGAACCTAGAAATCATACGCGGCAGGACCAAGCAACATGGTCAG

F S L A V V S L N I T S L G L R S L K E I

TTTTCTCTTGCAGTCGTCAGCCTGAACATAACATCCTTGGGATTACGCTCCCTCAAGGAGATA

S D G D V I I S G N A N L C Y A N T I N W

AGTGATGGAGATGTGATAATTTCAGGAAACGCGAATTTGTGCTATGCAAATACAATAAACTGG

K K L F G T S G Q K T K I I S N R G E N S

AAAAAACTGTTTGGGACCTCCGGTCAGAAAACCAAAATTATAAGCAACAGAGGTGAAAACAGC

C K A T G Q V C H A L C S P E G C W G P E

TGCAAGGCCACAGGCCAGGTCTGCCATGCCTTGTGCTCCCCCGAGGGCTGCTGGGGCCCGGAG

P R D C V S C R N V S R G R E C V D K V D

CCCAGGGACTGCGTCTCTTGCCGGAATGTCAGCCGAGGCAGGGAATGCGTGGACAAGGTCGAC

D P G E Q K L I S E E D L N G D P A K A A

GATCCGGGGGAACAAAAACTCATCTCAGAAGAGGATCTGAATGGAGATCCCGCAAAAGCGGCC

F D S L Q A S A T E Y I G Y A W A M V V V

TTTGACTCCCTGCAAGCCTCAGCGACCGAATATATCGGTTATGCGTGGGCGATGGTTGTTGTC

I V G A T I G I K L F K K F T S K A S

ATTGTCGGCGCAACTATCGGTATCAAGCTGTTTAAGAAATTCACCTCGAAAGCAAGC

- Sequence 12: HER-1 domain III **K443T**

M K K I W L A L A G L V L A F S A S A A E

ATGAAAAAGATTTGGCTGGCGCTGGCTGGTTTAGTTTTAGCGTTTAGCGCAAGTGCAGCTGAG

G A A A K V C N G I G I G E F K D S L S I

GGTGCGGCCGCCAAAGTGTGTAACGGAATAGGTATTGGTGAATTTAAAGACTCACTCTCCATA

N A T N I K H F K N C T S I S G D L H I L

AATGCTACGAATATTAAACACTTCAAAAACTGCACCTCCATCAGTGGCGATCTCCACATCCTG

P V A F R G D S F T H T P P L D P Q E L D

CCGGTGGCATTTAGGGGTGACTCCTTCACACATACTCCTCCTCTGGATCCACAGGAACTGGAT

I L K T V K E I T G F L L I Q A W P E N R

ATTCTGAAAACCGTAAAGGAAATCACAGGGTTTTTGCTGATTCAGGCTTGGCCTGAAAACAGG

T D L H A F E N L E I I R G R T K Q H G Q

ACGGACCTCCATGCCTTTGAGAACCTAGAAATCATACGCGGCAGGACCAAGCAACATGGTCAG

F S L A V V S L N I T S L G L R S L K E I

TTTTCTCTTGCAGTCGTCAGCCTGAACATAACATCCTTGGGATTACGCTCCCTCAAGGAGATA

S D G D V I I S G N T N L C Y A N T I N W

AGTGATGGAGATGTGATAATTTCAGGAAACACCAATTTGTGCTATGCAAATACAATAAACTGG

K K L F G T S G Q K T K I I S N R G E N S

AAAAAACTGTTTGGGACCTCCGGTCAGAAAACCAAAATTATAAGCAACAGAGGTGAAAACAGC

C K A T G Q V C H A L C S P E G C W G P E

TGCAAGGCCACAGGCCAGGTCTGCCATGCCTTGTGCTCCCCCGAGGGCTGCTGGGGCCCGGAG

P R D C V S C R N V S R G R E C V D K V D

CCCAGGGACTGCGTCTCTTGCCGGAATGTCAGCCGAGGCAGGGAATGCGTGGACAAGGTCGAC

D P G E Q K L I S E E D L N G D P A K A A

GATCCGGGGGAACAAAAACTCATCTCAGAAGAGGATCTGAATGGAGATCCCGCAAAAGCGGCC

F D S L Q A S A T E Y I G Y A W A M V V V

TTTGACTCCCTGCAAGCCTCAGCGACCGAATATATCGGTTATGCGTGGGCGATGGTTGTTGTC

I V G A T I G I K L F K K F T S K A S

ATTGTCGGCGCAACTATCGGTATCAAGCTGTTTAAGAAATTCACCTCGAAAGCAAGC

- Sequence 13: HER-1 domain III **I467M**

M K K I W L A L A G L V L A F S A S A A E

ATGAAAAAGATTTGGCTGGCGCTGGCTGGTTTAGTTTTAGCGTTTAGCGCAAGTGCAGCTGAG

G A A A K V C N G I G I G E F K D S L S I

GGTGCGGCCGCCAAAGTGTGTAACGGAATAGGTATTGGTGAATTTAAAGACTCACTCTCCATA

N A T N I K H F K N C T S I S G D L H I L

AATGCTACGAATATTAAACACTTCAAAAACTGCACCTCCATCAGTGGCGATCTCCACATCCTG

P V A F R G D S F T H T P P L D P Q E L D

CCGGTGGCATTTAGGGGTGACTCCTTCACACATACTCCTCCTCTGGATCCACAGGAACTGGAT

I L K T V K E I T G F L L I Q A W P E N R

ATTCTGAAAACCGTAAAGGAAATCACAGGGTTTTTGCTGATTCAGGCTTGGCCTGAAAACAGG

T D L H A F E N L E I I R G R T K Q H G Q

ACGGACCTCCATGCCTTTGAGAACCTAGAAATCATACGCGGCAGGACCAAGCAACATGGTCAG

F S L A V V S L N I T S L G L R S L K E I

TTTTCTCTTGCAGTCGTCAGCCTGAACATAACATCCTTGGGATTACGCTCCCTCAAGGAGATA

S D G D V I I S G N A N L C Y A N T I N W

AGTGATGGAGATGTGATAATTTCAGGAAACGCGAATTTGTGCTATGCAAATACAATAAACTGG

K K L F G T S G Q K T K I M S N R G E N S

AAAAAACTGTTTGGGACCTCCGGTCAGAAAACCAAAATTATGAGCAACAGAGGTGAAAACAGC

C K A T G Q V C H A L C S P E G C W G P E

TGCAAGGCCACAGGCCAGGTCTGCCATGCCTTGTGCTCCCCCGAGGGCTGCTGGGGCCCGGAG

P R D C V S C R N V S R G R E C V D K V D

CCCAGGGACTGCGTCTCTTGCCGGAATGTCAGCCGAGGCAGGGAATGCGTGGACAAGGTCGAC

D P G E Q K L I S E E D L N G D P A K A A

GATCCGGGGGAACAAAAACTCATCTCAGAAGAGGATCTGAATGGAGATCCCGCAAAAGCGGCC

F D S L Q A S A T E Y I G Y A W A M V V V

TTTGACTCCCTGCAAGCCTCAGCGACCGAATATATCGGTTATGCGTGGGCGATGGTTGTTGTC

I V G A T I G I K L F K K F T S K A S

ATTGTCGGCGCAACTATCGGTATCAAGCTGTTTAAGAAATTCACCTCGAAAGCAAGC

- Sequence 14: HER-1 domain III **S468R**

M K K I W L A L A G L V L A F S A S A A E

ATGAAAAAGATTTGGCTGGCGCTGGCTGGTTTAGTTTTAGCGTTTAGCGCAAGTGCAGCTGAG

G A A A K V C N G I G I G E F K D S L S I

GGTGCGGCCGCCAAAGTGTGTAACGGAATAGGTATTGGTGAATTTAAAGACTCACTCTCCATA

N A T N I K H F K N C T S I S G D L H I L

AATGCTACGAATATTAAACACTTCAAAAACTGCACCTCCATCAGTGGCGATCTCCACATCCTG

P V A F R G D S F T H T P P L D P Q E L D

CCGGTGGCATTTAGGGGTGACTCCTTCACACATACTCCTCCTCTGGATCCACAGGAACTGGAT

I L K T V K E I T G F L L I Q A W P E N R

ATTCTGAAAACCGTAAAGGAAATCACAGGGTTTTTGCTGATTCAGGCTTGGCCTGAAAACAGG

T D L H A F E N L E I I R G R T K Q H G Q

ACGGACCTCCATGCCTTTGAGAACCTAGAAATCATACGCGGCAGGACCAAGCAACATGGTCAG

F S L A V V S L N I T S L G L R S L K E I

TTTTCTCTTGCAGTCGTCAGCCTGAACATAACATCCTTGGGATTACGCTCCCTCAAGGAGATA

S D G D V I I S G N A N L C Y A N T I N W

AGTGATGGAGATGTGATAATTTCAGGAAACGCGAATTTGTGCTATGCAAATACAATAAACTGG

K K L F G T S G Q K T K I I R N R G E N S

AAAAAACTGTTTGGGACCTCCGGTCAGAAAACCAAAATTATACGTAACAGAGGTGAAAACAGC

C K A T G Q V C H A L C S P E G C W G P E

TGCAAGGCCACAGGCCAGGTCTGCCATGCCTTGTGCTCCCCCGAGGGCTGCTGGGGCCCGGAG

P R D C V S C R N V S R G R E C V D K V D

CCCAGGGACTGCGTCTCTTGCCGGAATGTCAGCCGAGGCAGGGAATGCGTGGACAAGGTCGAC

D P G E Q K L I S E E D L N G D P A K A A

GATCCGGGGGAACAAAAACTCATCTCAGAAGAGGATCTGAATGGAGATCCCGCAAAAGCGGCC

F D S L Q A S A T E Y I G Y A W A M V V V

TTTGACTCCCTGCAAGCCTCAGCGACCGAATATATCGGTTATGCGTGGGCGATGGTTGTTGTC

I V G A T I G I K L F K K F T S K A S

ATTGTCGGCGCAACTATCGGTATCAAGCTGTTTAAGAAATTCACCTCGAAAGCAAGC

- Sequence 15: HER-2 domain IV **D560A**

M K K I W L A L A G L V L A F S A S A A E

ATGAAAAAGATTTGGCTGGCGCTGGCTGGTTTAGTTTTAGCGTTTAGCGCAAGTGCAGCTGAG

G A A A C S Q F L R G Q E C V E E C R V L

GGTGCGGCCGCCTGCAGCCAGTTCCTTCGGGGCCAGGAGTGCGTGGAGGAATGCCGAGTACTG

Q G L P R E Y V N A R H C L P C H P E C Q

CAGGGGCTCCCCAGGGAGTATGTGAATGCCAGGCACTGTTTGCCGTGCCACCCTGAGTGTCAG

P Q N G S V T C F G P E A A Q C V A C A H

CCCCAGAATGGCTCAGTGACCTGTTTTGGACCGGAGGCTGCGCAGTGTGTGGCCTGTGCCCAC

Y K D P P F C V A R C P S G V K P D L S Y

TATAAGGACCCTCCCTTCTGCGTGGCCCGCTGCCCCAGCGGTGTGAAACCTGACCTCTCCTAC

M P I W K F P D E E G A C Q P C P I N C T

ATGCCCATCTGGAAGTTTCCAGATGAGGAGGGCGCATGCCAGCCTTGCCCCATCAACTGCACC

H S C V D L D D K G C P A E V D D P G E Q

CACTCCTGTGTGGACCTGGATGACAAGGGCTGCCCCGCCGAGGTCGACGATCCGGGGGAACAA

K L I S E E D L N G D P A K A A F D S L Q

AAACTCATCTCAGAAGAGGATCTGAATGGAGATCCCGCAAAAGCGGCCTTTGACTCCCTGCAA

A S A T E Y I G Y A W A M V V V I V G A T

GCCTCAGCGACCGAATATATCGGTTATGCGTGGGCGATGGTTGTTGTCATTGTCGGCGCAACT

I G I K L F K K F T S K A S

ATCGGTATCAAGCTGTTTAAGAAATTCACCTCGAAAGCAAGC

- Sequence 16: HER-2 domain IV **D560N**

M K K I W L A L A G L V L A F S A S A A E

ATGAAAAAGATTTGGCTGGCGCTGGCTGGTTTAGTTTTAGCGTTTAGCGCAAGTGCAGCTGAG

G A A A C S Q F L R G Q E C V E E C R V L

GGTGCGGCCGCCTGCAGCCAGTTCCTTCGGGGCCAGGAGTGCGTGGAGGAATGCCGAGTACTG

Q G L P R E Y V N A R H C L P C H P E C Q

CAGGGGCTCCCCAGGGAGTATGTGAATGCCAGGCACTGTTTGCCGTGCCACCCTGAGTGTCAG

P Q N G S V T C F G P E A N Q C V A C A H

CCCCAGAATGGCTCAGTGACCTGTTTTGGACCGGAGGCTAACCAGTGTGTGGCCTGTGCCCAC

Y K D P P F C V A R C P S G V K P D L S Y

TATAAGGACCCTCCCTTCTGCGTGGCCCGCTGCCCCAGCGGTGTGAAACCTGACCTCTCCTAC

M P I W K F P D E E G A C Q P C P I N C T

ATGCCCATCTGGAAGTTTCCAGATGAGGAGGGCGCATGCCAGCCTTGCCCCATCAACTGCACC

H S C V D L D D K G C P A E V D D P G E Q

CACTCCTGTGTGGACCTGGATGACAAGGGCTGCCCCGCCGAGGTCGACGATCCGGGGGAACAA

K L I S E E D L N G D P A K A A F D S L Q

AAACTCATCTCAGAAGAGGATCTGAATGGAGATCCCGCAAAAGCGGCCTTTGACTCCCTGCAA

A S A T E Y I G Y A W A M V V V I V G A T

GCCTCAGCGACCGAATATATCGGTTATGCGTGGGCGATGGTTGTTGTCATTGTCGGCGCAACT

I G I K L F K K F T S K A S

ATCGGTATCAAGCTGTTTAAGAAATTCACCTCGAAAGCAAGC
